# Modular Approach to Therapy for Anxiety, Depression, Trauma, or Conduct Problems in outpatient child and adolescent mental health services in New Zealand: study protocol for a randomized controlled trial

**DOI:** 10.1186/s13063-015-0982-9

**Published:** 2015-10-12

**Authors:** Mathijs F. G. Lucassen, Karolina Stasiak, Sue Crengle, John R. Weisz, Christopher M. A. Frampton, Sarah Kate Bearman, Ana M. Ugueto, Jennifer Herren, Ainsleigh Cribb-Su’a, Monique Faleafa, Denise Kingi-’Ulu’ave, Jik Loy, Rebecca M. Scott, Morgyn Hartdegen, Sally N. Merry

**Affiliations:** Department of Health and Social Care, The Open University, Walton Hall, Milton Keynes, MK7 6AA UK; Department of Psychological Medicine, School of Medicine, Level 12 Auckland City Hospital Support Building, University of Auckland, Private Bag 92019, Auckland, 1142 New Zealand; Invercargill Medical Centre, 160 Don Street, Invercargill, New Zealand; Department of Psychology, Harvard University, 1030 William James Hall, 33 Kirkland Street, Cambridge, MA 02138 USA; Department of Psychological Medicine, University of Otago (Christchurch), 2 Riccarton Avenue, PO Box 4345, Christchurch, 8140 New Zealand; Department of Educational Psychology, The University of Texas at Austin, 1912 Speedway, Stop D5800, Austin, TX 78712-1289 USA; Private clinical psychologist, Houston, USA; Department of Psychiatry and Human Behavior, Brown University, Box G-BH, Providence, RI 02912 USA; Whirinaki, Counties Manukau District Health Board, PO Box 217198, Botany Junction, Auckland 2164 New Zealand; Le Va, PO Box 76536, Manukau City, Auckland 2241 New Zealand; Infant, Child, and Adolescent Mental Health Services, Waikato District Health Board, Pembroke Street, Private Bag 3200, Hamilton, 3240 New Zealand

**Keywords:** adolescent, anxiety, child, conduct problems, depression, disruptive behavioral disorder, effectiveness, evidence-based treatments, post-traumatic stress disorder, randomized controlled trial

## Abstract

**Background:**

Mental health disorders are common and disabling for young people because of the potential to disrupt key developmental tasks. Implementation of evidence-based psychosocial therapies in New Zealand is limited, owing to the inaccessibility, length, and cost of training in these therapies. Furthermore, most therapies address one problem area at a time, although comorbidity and changing clinical needs commonly occur in practice. A more flexible approach is needed. The Modular Approach to Therapy for Children with Anxiety, Depression, Trauma, or Conduct Problems (MATCH-ADTC) is designed to overcome these challenges; it provides a range of treatment modules addressing different problems, within a single training program. A clinical trial of MATCH-ADTC in the USA showed that MATCH-ADTC outperformed usual care and standard evidence-based treatment on several clinical measures. We aim to replicate these findings and evaluate the impact of providing training and supervision in MATCH-ADTC to: (1) improve clinical outcomes for youth attending mental health services; (2) increase the amount of evidence-based therapy content; (3) increase the efficiency of service delivery.

**Methods:**

This is an assessor-blinded multi-site effectiveness randomized controlled trial. Randomization occurs at two levels: (1) clinicians (≥60) are randomized to intervention or usual care; (2) youth participants (7–14 years old) accepted for treatment in child and adolescent mental health services (with a primary disorder that includes anxiety, depression, trauma-related symptoms, or disruptive behavior) are randomly allocated to receive MATCH-ADTC or usual care. Youth participants are recruited from ‘mainstream’, Māori-specific, and Pacific-specific child and adolescent mental health services. We originally planned to recruit 400 youth participants, but this has been revised to 200 participants. Centralized computer randomization ensures allocation concealment. The primary outcome measures are: (i) the difference in trajectory of change of clinical severity between groups (using the parent-rated Brief Problem Monitor); (ii) clinicians’ use of evidence-based treatment procedures during therapy sessions; (iii) total time spent by clinicians delivering therapy.

**Discussion:**

If MATCH-ADTC demonstrates effectiveness it could offer a practical efficient method to increase access to evidence-based therapies, and improve outcomes for youth attending secondary care services.

**Trial registration:**

Australian and New Zealand Clinical Trials Registry ACTRN12614000297628.

## Background

Mental health problems are common in children and adolescents (youth) and the impact is considerable [1–5], with an estimated 50 % of all adult mental health disorders having an onset in adolescence [6]. A number of evidence-based therapies exist for the most common youth mental health problems, namely anxiety, depression, trauma-related symptoms, and disruptive behavior [1]. The Ministry of Health in New Zealand has repeatedly asserted the need for evidence-based treatments in mental health [7–9], including in child and adolescent mental health services [9]. There is evidence for the effectiveness of cognitive behavioral therapy for anxiety, depression, and trauma-related symptoms, while behavioral parent training is the treatment of choice for disruptive behavior [10–13]. Although these therapies have been shown to be effective in research settings, they are challenging to implement in clinical practice [14, 15]. For instance, most traditional evidence-based therapies focus on one disorder (or a small cluster of related disorders), making it difficult for clinicians to address heterogeneous caseloads, client comorbidities, and changes in clinical presentation during therapy [16]; training for each single-focus therapy can be time consuming and costly; and research participants in randomized controlled trials are typically treated for one disorder (whereas in clinical practice comorbidity is common).

New Zealand’s Child and Adolescent Mental Health Services (CAMHS) provide secondary level services for the 3–5 % of those with the most severe mental health problems aged 0–19 years [17]. The two largest professional groups employed in these services are nurses and social workers [18]; however, their pre-registration courses do not include training in psychotherapies specifically for youth [19]. Therefore, most learning occurs ‘on the job’. Furthermore, for clinicians to effectively treat a diverse caseload, they would need to train in multiple therapies, which is expensive and takes several years. For example, training in cognitive behavioral therapy is available in New Zealand in a one-year postgraduate course, which can be accessed by up to 12 clinicians per year. Accredited training in ‘Incredible Years’ (a form of evidence-based parent management training [20]) takes a year to complete and can be accessed by about 35 clinicians per year. Little training is available outside these courses and what exists will reach only a small minority of the approximately 1,000 full-time equivalent CAMHS clinicians in New Zealand [18]. The extent to which evidence-based therapies are currently used in CAMHS is not known, but based on clinicians’ access to training, and from overseas research [21–23], it is likely to vary across services and be limited by staff members’ past training, and may not be acceptable or effective for Māori (the indigenous people of New Zealand) or Pacific (people from the Pacific region) populations. Moreover, to meet the projected doubling of the demand on mental health services by 2020, treatment needs to be delivered more effectively and efficiently [24].

The Modular Approach to Therapy for Children with Anxiety, Depression, Trauma, or Conduct Problems (MATCH-ADTC) is a treatment system designed to work in day-to-day practice across a range of clinical problems [25]. MATCH-ADTC was developed following a number of meta-analyses of therapies with the best evidence of effectiveness [15, 26, 27] and following efforts to identify frequently used common elements of evidence-based practices for children and adolescents [28]. MATCH-ADTC was specifically designed to combine common elements of treatments for anxiety, depression, trauma-related symptoms, and disruptive behavior in one protocol, cater for comorbidity, and provide an opportunity to address fluctuations in presenting symptoms that might emerge during therapy [25, 29]. MATCH-ADTC comprises modules (i.e., specific practice elements) that can be organized in a flexible manner. Clinicians are guided by a MATCH-ADTC expert and an evidence-based algorithm to tailor treatment to each youth’s characteristics and needs. Youths and their families are also given an integral role in defining the goals of therapy. Furthermore, clinicians use a web-based system to monitor youths’ progress and adapt therapy until a problem is resolved. MATCH-ADTC has been evaluated in a randomized controlled trial (*n* = 174) comparing it against standard (single-focus) evidence-based therapies and usual care in the USA. The results showed that MATCH-ADTC was significantly more effective than standard evidence-based therapies and usual care, with effect sizes of 0.59–0.71 on primary outcome variables [30]. Furthermore, superiority relative to usual care was maintained in two-year follow-up analyses [31].

Our objective is to improve the overall quality of care received by youth attending CAMHS in New Zealand, by demonstrating improved clinical outcomes, increased efficiency of services, and increased delivery of evidence-based therapy. We have used Donabedian’s framework of structure, process, and outcome [32] to determine the potential impact of delivering MATCH-ADTC in New Zealand. In this study, our focus is on outcomes and process. We designed this trial to assess the effectiveness of MATCH-ADTC in the New Zealand context and we aimed in particular to investigate the acceptability and effectiveness for Māori and Pacific people. New Zealand has a growing Māori and Pacific population [33, 34], who are at increased risk of mental health problems [35–40], yet seldom specifically included in randomized controlled trials. In New Zealand, mental health services are delivered through ‘mainstream’ services. In some areas with large Māori and Pacific communities, there are specialized Kaupapa Māori and Pacific clinics (services that use delivery frameworks that are based on Māori or Pacific philosophies, values, and cultural practices).

This trial will contribute to the international literature by: (a) testing the effectiveness of MATCH-ADTC in publicly funded mental health services (a healthcare delivery model that is different from that used in the USA); and (b) providing information about the effectiveness of MATCH-ADTC across different ethnic groups.

### Primary hypotheses

We hypothesize that training CAMHS clinicians in MATCH-ADTC compared with usual care will:

i. Improve clinical outcomes for children and adolescents accessing CAMHS (outcomes); we will determine this by comparing the trajectories of change in clinical severity for the MATCH-ADTC and usual care groups (i.e., determining which group improves more quickly).
ii. Increase the delivery of evidence-based therapy (proces*s*); this will be measured by assessing the evidence-based content of recorded therapy sessions.
iii. Yield equal or better efficiency of service delivery (process); this will be measured by clinician time (in minutes) to deliver therapy, and duration (in weeks) of contact with the service by children or adolescents, and their families.

## Methods

### Design

This study is a multi-site effectiveness randomized controlled trial comparing MATCH-ADTC with usual care [41]. Prior to randomization, written informed consent is obtained from each participant. Randomization is implemented on two levels: first, randomization of participating clinicians, then randomization of youth participants. For the randomization of clinicians, all clinicians from participating services were invited to participate and provide written informed consent. Consenting clinicians were block randomized (by service or team) in a 1:1 ratio to MATCH-ADTC or usual care. The block size varied across sites, depending on the number of individual clinicians recruited from each site. Clinicians were stratified on the basis of previous training in evidence-based therapies (i.e., those with versus those without training in cognitive behavioral therapy or behavioral parent training). Clinicians randomized to MATCH-ADTC received training in MATCH-ADTC at the beginning of the study prior to the recruitment of youth participants. Those randomized to usual care will receive training at the end of the study (once all follow-up data have been collected). Randomization of youth participants occurs after a clinician-administered eligibility check and once written consent has been obtained. Youth participants are then randomized in a 1:1 ratio to receive MATCH-ADTC or usual care, with participants stratified by sex and ethnicity (Māori, Pacific, or ‘other’). Web-based randomization procedures are used to determine treatment allocation. Data from all participants will be included in data analysis, irrespective of whether or not all assessments have been completed.

### Changes to design during the trial

Two changes to the protocol were made during the course of the study.

1. As recruitment was lower than planned, particularly for Māori and Pacific clinicians and participants, it became clear that we would not have sufficient numbers to allow for useful statistical analysis by ethnicity. As a consequence we have reduced the number of planned analyses by ethnicity and adjusted our sample size accordingly. More details are provided in the ‘Sample size’ section. However, we plan to augment the data we will have on the use of MATCH-ADTC for these two populations with a qualitative study of the acceptability of MATCH-ADTC to Māori and Pacific clinicians, participants, and their families.
2. At the request of the Health Research Council’s independent data monitoring committee, who are overseeing the conduct and safety of the trial, we added to the data collection on serious adverse events by collecting information from parents at the three month follow-up interview on ‘moderate’ adverse events. More details on this are given below.

### Ethics approval

Approval has been received from New Zealand’s Health and Disability Ethics Committee (13/CEN/97).

### Setting

This study is being conducted in ten outpatient CAMHS clinics from four provinces in New Zealand (Northland, Auckland, Waikato, and Wellington).

### Participants

Participants are youths and their parents (or guardians), referred to CAMHS for assessment and treatment. Intake clinicians identify potential participants during their standard initial assessment. One self-nominated parent completes the parent-rated assessments.

### Inclusion criteria

Participants are eligible if:

- They are newly referred to, and accepted for treatment in, CAMHS with a primary disorder that includes anxiety, depression, trauma-related symptoms, or disruptive behavior;
- They are 7 to 14 years of age on the date of consent;
- They provide written consent (or verbal assent) and have written parental consent; and,
- They and their parents speak English.

### Exclusion criteria

Participants are ineligible if:

- They are currently receiving other psychosocial treatment for their disorder within or outside CAMHS;
- They have a primary disorder of psychosis, intellectual disability, attention deficit-hyperactivity disorder (where the primary reason for referral is inattention or over-activity), autism, or an eating disorder;
- They are acutely suicidal; or,
- They have a sibling who has previously been recruited into the study.

### Withdrawal criteria

Participants (youth and their parents, or study clinicians) can withdraw from the study at any time. Those participants who remain in the study but have dropped out of treatment are followed up wherever possible. Where a clinician withdraws from the study, a clinician from the same treatment arm will continue to provide the treatment allocated (i.e., either MATCH-ADTC or usual care).

### Intervention

#### Intervention (MATCH-ADTC)

MATCH-ADTC consists of a manual, a training package, and a monitoring and feedback system (the eMonitor in this study). Clinicians participated in an initial five day training of block teaching, as well as weekly small group telephone or Skype consultation sessions, led by an accredited MATCH-ADTC expert to support ongoing fidelity to the model. MATCH-ADTC comprises 33 modules or specific treatment procedures. Clinicians delivering MATCH-ADTC follow structured and specific treatment procedures, as outlined in the MATCH-ADTC manual. At the start of treatment, a clinician meets with the youth participant and the youth’s family and they collaboratively establish top problems to be addressed in therapy, thus providing the participant and their family an integral role in defining the goals for therapy. Following this, clinicians (with the support of a MATCH-ADTC expert) are guided by an evidence-based algorithm to ensure that treatment is best tailored to the youth’s clinical presentation. Each MATCH-ADTC module contains details about the therapy session, including such resources as therapy worksheets, homework assignments, and caregiver handouts. Clinicians use a web-based system (the eMonitor, designed purposefully for this trial), which provides a weekly summary of the participant’s progress (based on the results of the Brief Problem Monitor and Top Problem Assessment), to monitor their client’s progress and adapt therapy in consultation with the youth and the youth’s family.

#### Intervention fidelity

MATCH-ADTC therapists have weekly phone or Skype supervision where treatment for each youth is reviewed. Clinicians follow the MATCH-ADTC manual with the support of a MATCH-ADTC expert. All sessions (MATCH-ADTC and usual care) are audio-recorded and a randomly selected subset will be coded for evidence-based therapy content.

#### Control (usual care)

Usual care is the treatment that is usually provided to a youth at a CAMHS (e.g., case management, individual therapy, family therapy, medication, psycho-education, or a combination of these). Information on the usual care provided to each participant is collected. Clinicians providing usual care do not have access to the weekly progress data on the eMonitor, as this is not part of usual care.

Clinicians providing both MATCH-ADTC and usual care receive support from their team leaders and clinical managers, but MATCH-ADTC clinicians are instructed not to discuss with the rest of their team the treatment approaches they are using with their MATCH-ADTC clients, to ensure there is no contamination. This approach was used successfully in the study in the USA [30].

### Outcomes and measures

#### Primary outcomes

The three primary outcomes each address one of the three central aims of the trial: (i) the difference in trajectory of change of clinical severity between groups, using the parent-rated Brief Problem Monitor [42]; (ii) clinicians’ use of evidence-based content during therapy sessions (based on audio-recorded therapy sessions); (iii) total time spent by clinicians delivering therapy (using therapy logs).

#### Secondary outcomes

Other clinical outcomes are measured using: the youth-rated Brief Problem Monitor [42], the parent- and youth-rated Strengths and Difficulties Questionnaire [43], the parent- and youth-rated Top Problems Assessment [44], the youth-rated Child Health Utility [45], the number and type of diagnoses using the Development and Well-Being Assessment [46, 47], prescribed medications (and dosage) for psychiatric conditions.

Further secondary outcomes include:

- Clinician self-report of the treatment provided based on the Therapy Procedures Checklist [48];
- Clinician’s satisfaction with therapy based on the Therapist Satisfaction Inventory [49];
- Client satisfaction with therapy based on a treatment satisfaction questionnaire;
- The number and types of serious adverse events and moderate adverse events.

### Outcome measures

The following outcome measures are collected during the trial. Most clinical measures are collected during the weekly telephone calls made by the research assistants according to the schedule of assessments listed in Table 1.

**Table 1:** Summary schedule of data collected

Measure	Baseline	During therapy	After intervention	3-month follow-up	Informant	Collected by
Demographics	×				Child and parent	Clinician
Strengths and Difficulties Questionnaire	×	monthly	×	×	Child and parent	Research assistant
Brief Problem Monitor	×	weekly	×	×	Child and parent	Research assistant
Top Problems Assessment	×	weekly	×	×	Child and parent	Clinician at baseline and research assistant weekly
Development and Well-Being Assessment	×		×		Child (if 11 or over) and parent	Research assistant supports the family to complete online
Child Health Utility	×		×	×	Child	Research assistant
Medication use	×		×	×	Clinician and parent	Clinician at baseline and after intervention, research assistant at 3 months
Client satisfaction			×		Child and parent	Research assistant
Therapy Procedures Checklist			×		Clinician	Clinician
Serious adverse events		×			Clinician	Clinician
Moderate adverse events				×	Parent	Research assistant
Therapist Satisfaction Inventory			×		Clinician	Clinician
Recordings of therapy sessions		×			Clinician	Clinician
Therapy log			×		Clinician	Clinician

The Brief Problem Monitor [42] is a 19-item assessment using data from parents and youth to measure internalizing, externalizing, hyperactivity, and total problems. It is based on the widely used Child Behavior Checklist [50] and Youth Self-Report [51], both of which have sound psychometric qualities. The Brief Problem Monitor can be administered by phone, taking approximately five minutes to complete per administration, making it a practical and robust measure of the trajectory of change in clinical symptoms over time.

- The Top Problems Assessment has been developed to allow the youth and family to identify and rate the severity of the three top problems that they would like addressed in therapy. There is good evidence for reliability, validity, and sensitivity to change for this assessment [44].
- The Strengths and Difficulties Questionnaire [43] is a scale used widely in both research and practice. It produces a total score with five subscales: emotional symptoms, conduct problems, hyperactivity, peer relationship problems, and prosocial behavior. It has a satisfactory internal consistency, test–retest reliability, and inter-rater agreement [52].
- Child Health Utility [45] is a nine-item measure of health-related quality of life designed specifically for children. Each item taps into a different domain (i.e., worry, sadness, pain, tiredness, annoyance, school, sleep, daily routine, and activities) in reference to how a child feels ‘today’. This is a preference-based instrument that generates utility weights and which allows for the calculation of quality-adjusted life years for use in health economic evaluations. The internal consistency has been reported sound and the convergent validity against the Strengths and Difficulties Questionnaire is in the moderate-to-strong range [53].
- The Development and Well-Being Assessment [46] is an online diagnostic tool. It is designed to indicate likely psychiatric diagnoses in 5–16-year-olds based on in-built diagnostic algorithms. This assessment has been validated in both community and clinic samples in the UK [46]. Inter-rater agreements between the Development and Well-Being Assessment and clinician diagnoses are moderate to high [54–56].
- Client satisfaction questionnaires. The parent-rated measure has nine questions (eight-rated on a Likert scale and one open-ended item). The youth-rated satisfaction questionnaire includes seven questions (four rated on a Likert scale and three open-ended items). Likert-rated questions ask the informant to provide feedback on the quality of treatment, whether it helped, and if they would recommend it to others. The open-ended questions were designed to elicit what the participants liked most or least about the treatment and how treatment could be improved.
- The Therapist Satisfaction Inventory is a 16-item questionnaire (adapted from [49]), which assesses how satisfied a clinician feels about the intervention provided.
- The Therapy Procedures Checklist is a treatment description questionnaire, which consists of 62 items describing common psychotherapeutic processes (i.e., psychodynamic, cognitive, and behavioral techniques) that may have been used in the course of therapy. Each item is rated on a five-point Likert scale from ‘rarely’ to ‘most of the time’. The instrument is adapted from an earlier version of the Therapy Procedures Checklist, which has been shown to have good content validity, internal consistency, and test–retest reliability [48].
- The medication use questionnaire collects data on prescribed medications (and dosage) for psychiatric conditions.

The efficiency of service delivery is assessed using the therapy log. It includes: (1) the date and duration of each therapy session (in minutes); (2) whether non-study clinicians were also involved in a session; (3) the location of a therapy session (e.g., clinic, home, school); and (4) whether any scheduled sessions were unattended by the youth and family.

The amount of evidence-based therapy content is based on digitally recorded therapy sessions (all sessions, both MATCH-ADTC and usual care). A randomly selected subset of 10 % of therapy sessions from each arm are assessed for evidence-based content by the research team (blind to treatment allocation) based on the methods and the coding system (Bearman S, Herren J, Weisz J., Therapist Integrity to Evidence-Based Interventions, 2012, unpublished manual) developed for the initial trial of MATCH-ADTC [30]. A subset of this sample is independently double-coded to confirm inter-rater agreement. The Therapist Integrity to Evidence-Based Interventions is a micro-analytic system for coding therapy sessions for the fidelity (adherence and competence) with which a therapist utilized therapeutic techniques used in MATCH-ADTC [25]. Scores on this measure reflect both adherence (frequency and thoroughness) and competency (skillfulness). The Therapist Integrity to Evidence-Based Interventions was adapted from a previous coding system, in order to merge overlapping items [30]. This version has shown to have excellent levels of coder agreement for both adherence (multivariate intraclass correlation coefficient = 0.84) and competence (multivariate intraclass correlation coefficient = 0.78) in a sample of community therapists in the USA delivering both MATCH-ADTC and usual care [57].

#### Measures of harm

A serious adverse event is defined as an event that: (1) results in the participant’s death, (2) is a suicide attempt; (3) results in hospitalization for non-suicidal self-harm; or (4) results in hospitalization for mental health problems. Clinicians are instructed to report serious adverse events at any stage during therapy within one working day of being aware of the event. A report of a serious adverse event is followed by a telephone call to the clinician from the research team to collect additional information on the serious adverse event, in order to determine the relationship to the study, and the appropriate course of action (continuation or withdrawal from the trial). All serious adverse events are reviewed by a senior clinician independent of the study and reported to the independent data monitoring committee (from the Health Research Council) and the trial steering committee.

Moderate adverse events are defined as: (1) hospitalization for any medical reason, other than mental health problems; (2) serious behavioral problem (i.e., suspension or expulsion from school, running away from home, or problems with the police); or, (3) the use of formal respite care. Information on moderate adverse events is recorded when reported during the study and also collected from the parents at follow-up interview. These events are also reported to the independent data monitoring committee (from the Health Research Council) and the trial steering committee.

### Assessment process

#### Screening for eligibility

Young people referred to CAMHS are provided with a routine assessment by an intake clinician. Potential participants who are deemed suitable for treatment at the service and meet study criteria are then invited to take part in the study. The assessing clinician obtains consent or assent from the youth and parent and collects the demographics data. Those who are eligible and have given consent are then randomized, and a study site coordinator allocates a clinician to provide therapy according to treatment group allocation.

#### Baseline measurement

Once the clinician has established with the youth and parent the key problems, using the Top Problems Assessment in a face-to-face session, weekly ratings on these are collected by the research assistant via telephone. Other baseline measures are collected by the research assistant.

#### Weekly measurements

The allocated research assistant completes the weekly telephone assessments with the parent and the youth participant for the duration of treatment. The weekly assessment is designed not to exceed 10 minutes, to minimize participant burden.

#### Monthly assessment

Once a month, in addition to the weekly measures, the research assistant conducts the Strengths and Difficulties Questionnaire (parent- and youth-rated) assessments.

#### Post-intervention assessment

The post-intervention assessment occurs once a clinician has discharged the youth from treatment and is conducted by the research assistant.

#### Three month follow-up

Three months after the post-intervention assessment, a final follow-up assessment is conducted by the research assistant.

**Fig. 1:**
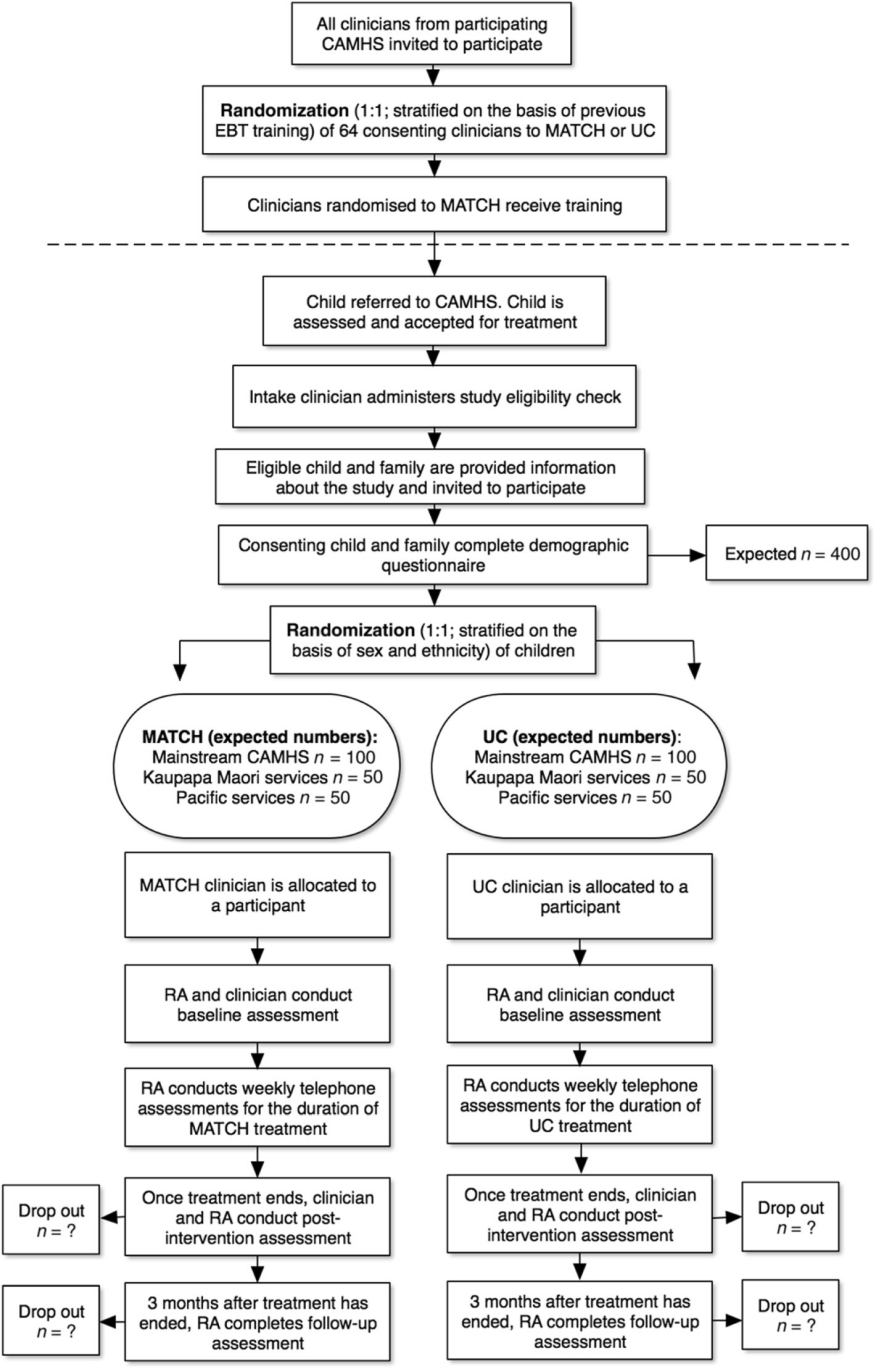
Participant flow. Note that this is the original flow chart and has been amended to 100 participants per arm with the reduced recruitment target. As many participants as possible are recruited from Kaupapa Māori and Pacific services. CAMHS, Child and Adolescent Mental Health Services; EBT, evidence-based treatment; MATCH, Modular Approach to Therapy for Children with Anxiety, Depression, Trauma, or Conduct; RA, research assistant; UC, usual care

Participant flow is outlined in Fig. 1. The study procedures are summarized in Table 1.

### Sample size

Overall, a sample size of 60 clinicians and 400 patients will provide sufficient power to detect effect sizes of approximately 0.37, well below the effect size shown by the comparable USA study [30]. The initial power calculation was to allow independent analysis to be undertaken for Māori and Pacific participants and ensured sufficient power (80 %) to detect moderate effect sizes of between 0.61 and 0.70 as statistically significant (two-tailed *α* = 0.05) in these groups (equivalent to the effect sizes found in the USA study [30]). This estimate allowed for a 15 % attrition rate in clinicians and in clients (based on the 12.3 % attrition rate reported in Weisz *et al.* [30] and adjustment for the clustering of patients with clinicians (intraclass coefficient = 0.1)). We assumed approximately six to eight patients per clinician (approximately 400 patients). If fewer clinicians were available within the targeted services, we planned to increase the number of participants per clinician to ensure that we maintained statistical power. The intention was to recruit approximately 12 clinicians working in Kaupapa Māori services and approximately 12 clinicians working in Pacific services. While this seemed feasible in the planning stages, when we were actually conducting the trial, there were a number of staffing difficulties in the services overall, and these impacted more particularly in the Kaupapa Māori and Pacific services, perhaps because of the smaller pool of people in the workforce. This led to difficulty in recruiting clinicians working in Māori and Pacific services, with resulting reduced ability to recruit participants to the trial from those services. It became evident that we would not reach the targets for Māori and Pacific participants to allow ethnic-specific analysis, as planned. An interim blinded-to-treatment allocation, single group, power analysis was undertaken (when approximately 50 participants had completed treatment) to determine the total number of participants needed to show overall clinical effectiveness for the whole sample. This summary produced a standard deviation for the change in the parent-rated Brief Problem Monitor of 4.4, which indicated that to show a clinically important difference in the change (>2 units) as statistically significant (two-tailed *α* = 0.05) with 80 % power, a total sample size of 160 participants would need to be recruited. A total sample of 200 participants would provide 90 % power for this comparison. Although a reduced final sample size would compromise the ethnic-specific analysis, it was felt that the primary question of whether MATCH-ADTC improved the trajectory of change of clinical severity more than usual care could be conclusively addressed with 200 participants. Based on the ongoing recruitment, it was anticipated that this total recruitment could be achieved if we extended the timeline and recruited until the end of June 2015.

### Randomization and sequence generation

Youth are recruited over many months, and are not all available for randomization at the outset of the trial, whereas the clinicians had to be randomized initially so that those in the MATCH-ADTC condition could all receive training prior to recruiting youth for the study. We believe that any possible selection bias has been avoided, as any youth participant who is randomized can then be immediately allocated to their randomization group. Because this random allocation was carried out by the study team, with no input from clinicians or clinic staff, there was no possibility of the screening or allocation process being biased by any of the treating clinicians. The randomization sequences for clinicians and youth participants were electronically generated in permuted blocks prior to any recruitment and loaded into the eMonitor system.

### Allocation concealment

Allocation concealment for the participants and researchers has been assured by using centralized computer allocation of the randomization sequence. The randomization list is not available to any members of the research team directly involved in the assessment or screening of participants. A participant is only randomized once all entry criteria are met. After the details of a consenting participant are entered online, the eMonitor informs the study site coordinator or the clinician of the randomized group allocation.

### Blinding

Owing to the nature of the study, double blinding is not possible, as clinicians are aware of the treatment they are administering. We anticipate that despite signing informed consent, most youth and their parents will effectively be blind to treatment allocation. At entry to the study, the information conveyed to the participants (orally and in writing) has been designed to ensure that details that could easily distinguish MATCH-ADTC from usual care are not provided, but that accurate information is given. We explain that both treatments are thought to be effective and that the purpose of this research is to compare treatments to see if one is better than the other. All research assistants collecting telephone administered assessments and coding the therapy sessions are blind to treatment allocation throughout the study. Participants are asked not reveal the details of the intervention they are receiving to the research assistant. All participants are given an identification number to ensure that the researchers involved in data management and manipulation are unaware of the treatment allocation.

### Statistical methods

#### Interim analysis

When recruitment was lower than anticipated, a single interim blind calculation of the standard deviation of the change in the primary outcome parent-rated Brief Problem Monitor for all participants as a single group was undertaken, to determine the utility of recruiting a smaller sample size than originally planned. As this summary did not involve a comparison between randomized groups, no adjustment to the *α* level for protection of the type I error rate was required.

#### Overall analysis

The comparability of the baseline status of both treatment groups (i.e., MATCH-ADTC and usual care) will be determined using descriptive analysis, in terms of age, sex, clinical symptom severity, diagnosis, and ethnicity. The two randomized treatments may vary in duration, so that post-intervention and follow-up (three months after treatment has ended) analyses are potentially confounded by treatment duration. To allow an unconfounded interpretation, we propose to use the trajectories of change across time on the parent-rated Brief Problem Monitor as our primary outcome measure. The trajectory of change was selected in part because the duration of treatment and number of sessions provided to individual youth are variable in usual clinical services, and also within the structure of the MATCH-ADTC program, such that the usual care and MATCH-ADTC groups could not be matched for duration or number of sessions. In addition, we want to compare the results of the current study with that of a trial from the USA that used this method [30]. The primary analysis will be based on the intention-to-treat population, but sensitivity analysis will be undertaken on the per-protocol population.

#### Primary outcomes

##### Clinical outcomes

The difference in the trajectories between groups will be tested using a mixed effects regression model: *a*_0_ (intercept) + *a*_1_ (participant) + *a*_2_ (treatment group) + *a*_3_ (time) + *a*_4_ (treatment × time) and intercept and participant as random effects. These analyses will further include terms for:

- Service type (i.e., Kaupapa Māori, Pacific and ‘mainstream’ CAMHS);
- Clinicians’ previous training in evidence-based therapy;
- Baseline symptom level; and
- Medication use.

Although we are now unable to conduct ethnic-specific analysis, summary data will be used to illustrate whether there is a broad consistency of the clinical outcome results across ethnicity groups.

##### Delivery of evidence-based treatments

The percentage of evidence-based content and quality of delivery of therapy in a 10 % random subset of sessions assessed by coding audio-taped therapy sessions will be compared between groups using an analysis of variance (ANOVA) model, following the method used in the trial in the USA study [30].

##### Efficiency of service delivery

Efficiency in delivery of therapy for MATCH-ADTC and usual care will be compared using ANOVA to test for significant differences using the following data which will be collected from the clinical service, including:

- Clinician time (in minutes);
- Duration of contact with the service (in weeks);
- Number of therapy sessions; and
- Number of missed therapy sessions.

If any of these outcomes do not meet the assumptions for parametric ANOVA models, the data will be transformed as appropriate prior to analysis.

## Discussion

This trial has been designed to balance the need to replicate the evaluation of MATCH-ADTC conducted in the USA with the objective of evaluating it in a New Zealand context. We anticipate that if results are positive, MATCH-ADTC may become part of core training for CAMHS clinicians, resulting in better outcomes for youth with mental health challenges, increase in delivery of evidence-based treatments, and improved efficiency of service delivery. This trial therefore has practical implications for the workforce, as MATCH-ADTC is the first comprehensive evidence-based system that could realistically become part of a ‘core competency’ training package for all CAMHS clinicians.

We selected brief measures to keep the weekly telephone assessments as brief as possible, and lessen participant burden. Frequent assessments allow for monitoring of change when treatment duration varies considerably and the end of treatment is unknown. We have used an online diagnostic tool because we do not have dedicated researchers at each site, and because formal diagnoses are not routinely obtained in each CAMHS in New Zealand. In return for the time taken to complete the assessment, and to maximize Development and Well-Being Assessment completion, we have chosen (with ethics committee approval) to incentivize families with gift vouchers. Each family received a NZ$30 (approximately US$20) supermarket gift voucher for completing the Development and Well-Being Assessment at baseline and a second NZ$30 voucher after intervention. Those who completed both the baseline and the post-intervention Development and Well-Being Assessment received an additional NZ$30 (in total NZ$90). These gift vouchers were provided as a gratuity for the time taken to complete these assessments.

Most CAMHS in New Zealand structure their services using the Choice and Partnership Approach [58], in which an intake clinician conducts the initial assessment to determine whether the treatment at CAMHS is suitable for the young person, and then a treatment clinician is allocated and therapy is provided by the allocated clinician. This allows for a screening assessment and for randomization at the intake session without disrupting the rapport established between the family and the clinician, as the allocated clinician usually differs from the intake clinician.

One of the practical challenges for this study is determining the end of treatment because services have different guidelines in place regarding discharge (e.g., a case can be left open for a number of weeks after a proposed final session, in case a youth and their family would like an extra ‘top-up’ session). If needed, the regular weekly assessments and the therapy log data will allow us to determine retrospectively when treatment ended and link this to the correct weekly telephone administered assessments. Another issue has been the recruitment of youths at Māori and Pacific sites. The challenges that impacted on recruitment of these groups included the smaller pool of clinicians at Kaupapa Māori and Pacific services, the smaller proportion of youths meeting study criteria because of high acuity of the problems (including suicidality) and the lower numbers of youths within our age range.

This is a pragmatic trial [41] and accordingly the exclusion criteria have been kept to a minimum and youth participants’ presentation (not their diagnoses) have determined eligibility for participation in the study. As such, we believe this study will have good external validity, because the participants reflect the heterogeneity of youth from ‘real-life’ clinical services. Hence the findings will be applicable to other CAMHS in New Zealand and will probably be of relevance to other countries that have a comparable healthcare workforce and service delivery models.

## Trial status

We are currently recruiting participants.

## Abbreviations

ANOVA: analysis of variance
CAMHS: Child and Adolescent Mental Health Services
MATCH-ADTC: Modular Approach to Therapy for Children with Anxiety, Depression, Trauma, and Conduct Problems.
